# Authors' Reply

**DOI:** 10.18502/jovr.v15i1.5964

**Published:** 2020-02-02

**Authors:** Zisis Gatzioufas, Mohamed Elalfy, Samer Hamada

**Affiliations:** ^1^Department of Ophthalmology, Corneo-Plastic Unit, Queen Victoria Hospital NHS Trust, East Grinstead, UK

Dear Editor,

We thank Srirampur and Balijepalli^[1]^ for their interest in our case report.

We completely agree with their important comment on the mechanism of hydrophilic intraocular lens opacification after keratoplasty, particularly following endothelial keratoplasty in which air or gas tamponade is performed.

However, we reported a case of hydrophobic intraocular lens opacification after penetrating keratoplasty, and to the best of our knowledge, this was the first time that this type of intraocular lens opacification occurred. Anterior segment surgeons should be aware of this rare complication, as advised by Srirampur and Balijepalli.
